# miRNA expression changes during the course of neoadjuvant bevacizumab and chemotherapy treatment in breast cancer

**DOI:** 10.1002/1878-0261.12561

**Published:** 2019-08-28

**Authors:** Evita Maria Lindholm, Miriam Ragle Aure, Mads Haugland Haugen, Kristine Kleivi Sahlberg, Vessela N. Kristensen, Daniel Nebdal, Anne‐Lise Børresen‐Dale, Ole Christian Lingjærde, Olav Engebraaten

**Affiliations:** ^1^ Department of Cancer Genetics, Institute for Cancer Research, The Norwegian Radium Hospital Oslo University Hospital Norway; ^2^ Department of Tumor biology, Institute for Cancer Research, The Norwegian Radium Hospital Oslo University Hospital Norway; ^3^ Department of Research and Innovation Vestre Viken Hospital Trust Drammen Norway; ^4^ Department of Clinical Molecular Biology (EpiGen), Division of Medicine Akershus University Hospital Lørenskog Norway; ^5^ Insitute for Clinical Medicine University of Oslo Norway; ^6^ Department of Computer Science, Faculty of Mathematics and Natural Sciences University of Oslo Norway; ^7^ Department of Oncology Oslo University Hospital Norway

**Keywords:** angiogenesis, bevacizumab, breast cancer, miRNA, neoadjuvant

## Abstract

One of the hallmarks of cancer is sustained angiogenesis. Favorable results have been reported in some breast cancer (BC) patients receiving antiangiogenic therapy with bevacizumab (Bev) in combination with chemotherapy, and further knowledge on how Bev can be optimally combined with conventional treatment to increase efficacy is strongly needed. In this randomized, neoadjuvant phase II clinical trial, 132 patients with HER2‐negative, nonmetastatic BC were treated with Bev in combination with sequential chemotherapy. Biopsies were sampled before treatment, after 12 weeks with anthracycline and after taxane therapy at week 25. MicroRNA (miRNA) expression profiling was performed on biopsies from each time point. Altogether, 241 biopsies were analyzed with the aim of identifying miRNA‐based biomarkers of response to therapy. Results from the miRNA analyses were reported for the ER‐positive cohort, which were previously demonstrated to benefit from antiangiogenic therapy in this study. For both treatment arms of this cohort, significantly different expression was observed for 217 miRNAs between objective responding and nonresponding patients before treatment initiation. These miRNAs have been linked to regulation of epithelial–mesenchymal transition, metastasis, and tumor growth, among other processes. Bev in combination with chemotherapy resulted in similar miRNA changes to chemotherapy alone. However, the deregulation of miRNA expression occurred earlier in the Bev arm. In both arms, tumor suppressor miRNAs were found upregulated after treatment, while oncogenic miRNAs were downregulated in the Bev arm. Patients responding to Bev showed a strong correlation between deregulated miRNAs and decreased proliferation score during the course of treatment, with downregulation of miR‐4465 as the strongest indicator of reduced proliferation. Integrative analyses at miRNA‐, gene‐, and protein expression further indicated a longitudinal decrease in proliferation. Altogether, the results indicate that proliferation might represent a predictive factor for increased Bev sensitivity, which may aid in the identification of patients who could potentially benefit from Bev.

**Abbreviations**
BC: breast cancer
EMT: epithelial–mesenchymal transition
ER: estrogen receptor
FDR: false discovery rate
IPA: ingenuity pathway analysis
miRNA: microRNA
OR: objective response
OS: overall survival
pCR: pathological complete response
PFS: progression‐free survival

## 1 Introduction

Among women, breast cancer (BC) is the most common cancer type worldwide and the leading cause of cancer death (Bray *et al.*, 2018). The mechanisms underlying BC development are diverse and complex, with the different molecular subtypes responding differentially to chemotherapy and targeted agents (Rouzier *et al.*, 2005).

Although new and improved treatments have resulted in a more than 80% 5‐year survival for BC patients, improved therapeutic strategies are still needed. Tumor angiogenesis represents one of the hallmarks in cancer, and differential expression of important mediators of this process, in particular vascular endothelial growth factor (*VEGF*), has been associated with tumor progression and poor prognosis in several tumor types, including BC (Berns *et al.*, 2003; Manders *et al.*, 2002). The anti‐VEGF antibody bevacizumab (Bev) has shown increased overall survival (OS) and progression‐free survival (PFS) in patients with colorectal cancer when combined with first‐line chemotherapy (Hurwitz *et al.*, 2013). In BC, treatment with Bev in combination with chemotherapy has shown less success, with variable improvements in PFS and no benefits in OS (Miles *et al.*, 2010; Miller *et al.*, 2007). One potential explanation for the variation in outcome may be due to differential response in different BC subtypes, as shown in two neoadjuvant clinical trials with Bev; one reported increased response rates in the triple negative BC subset (von Minckwitz *et al.*, 2012), whereas the other reported most responding patients in the estrogen receptor (ER)‐positive BC patients (Bear *et al.*, 2012). Accordingly, there is a need for a better understanding of the underlying molecular and biological mechanisms to enable early identification of BC patients responding to neoadjuvant antiangiogenic therapy. In addition to the heterogeneity of BC defined by the different transcriptomic subtypes, microRNAs (miRNAs) have been shown to increase this complexity through their posttranscriptional regulation of gene expression (Aure *et al.*, 2015; Aure *et al.*, 2017).

MicroRNAs have been identified as important regulators of cellular processes (Sun *et al.*, 2010) and have been demonstrated to have both oncogenic and tumor suppressor roles in different tumor types (Kent and Mendell, 2006; Kurozumi *et al.*, 2017). miRNAs have also been shown to have pro‐angiogenic effects (i.e., the miR‐17‐92 cluster (Suarez *et al.*, 2008), miR‐21 (Liu *et al.*, 2011), and miR‐378 (Lee *et al.*, 2007)) or antiangiogenic effects (i.e., miR‐221 and miR‐222 (Poliseno *et al.*, 2006)). Studies in colorectal cancer, glioblastoma, and ovarian cancer have identified miRNAs with potential as predictors for antiangiogenic therapy by comparing expression levels in responders vs. nonresponders in surgical resections from the primary tumor before or after treatment (Chan *et al.*, 2014; Hayes *et al.*, 2016; Kiss *et al.*, 2017). Changes in circulating miRNAs have also been assessed, where miR‐126 has been shown to predict response in patients with metastatic colorectal cancer during chemotherapy and Bev treatment (Hansen *et al.*, 2015). For BC, few studies have elucidated the role of miRNAs in response to Bev response. One report indicates that low expression of miR‐20a‐5p in BC patients is associated with response to Bev (Gampenrieder *et al.*, 2017), but to our knowledge, the present study is the first to describe concerted changes in miRNA expression related to Bev treatment and response in BC.

In the current study, patients with HER2‐negative breast carcinomas were randomized to neoadjuvant treatment with chemotherapy either alone or in combination with Bev. miRNA expression profiles from specimens taken before, during, and at the end of 25 weeks of treatment were compared to treatment response. Furthermore, important parameters like tumor proliferation, tumor stage, intrinsic subtype of BC classification (PAM50), epithelial‐to‐mesenchymal transition (EMT), and relapse were analyzed with respect to miRNA expression, resulting in the identification of potential candidate miRNAs involved in treatment response.

## 2 Materials and methods

### 2.1 Patient population and study design

Patient inclusion criteria have been described previously (Silwal‐Pandit *et al.*, 2017). The experiments were undertaken with the understanding and written consent of each subject. The study methodologies conformed to the standards set by the Declaration of Helsinki, and the study methodologies were approved by the local ethics committee. Briefly, patients with previously untreated, HER2‐negative, large (≥ 2.5 cm; stage T2, T3, or T4) mammary tumors were eligible for inclusion. A total of 138 patients were randomized 1 : 1 to receive either chemotherapy or chemotherapy in combination with Bev. The chemotherapy regimen in the study consisted of four cycles of FEC100 (five fluorouracil 600 mg·m^−2^, epirubicin 100 mg·m^−2^, and cyclophosphamide 600 mg·m^−2^) every 3 weeks, followed by four cycles of docetaxel 100 mg·m^−2^ every 3 weeks or 12 weekly infusions of paclitaxel 80 mg·m^−2^. Bev was administered intravenously at a dose of 10 mg·kg^−1^ every other week or 15 mg·kg^−1^ every third week in patients receiving paclitaxel or docetaxel, respectively. Core needle biopsies were collected before treatment (week 0), after completion of 12 weeks of treatment (week 12), and from the surgical specimen (week 25). Patients with ER‐positive and ER‐negative tumors were included in the study; however, only ER‐positive tumors were considered in the miRNA analyses described here, as these patients were shown to have a statistical significant benefit from adding Bev to the chemotherapy (Silwal‐Pandit *et al.*, 2017). For the ER‐positive tumors, miRNA expression profiles were available for 97 patients at week 0, 65 patients at week 12, and 79 patients at week 25.

### 2.2 Treatment response

According to the RECIST criteria (Eisenhauer *et al.*, 2009), partial response (PR) is defined as more than 30 % and < 100% decrease in the sum of the longest diameter of target lesions, while objective response (OR) is either PR or complete response. Therefore, we define a nonresponder as a patient with tumor shrinkage ≤ 30%. The number of samples from each time point from objective responding patients vs. nonresponding patients was 68 vs. 22 at week 0, 44 vs. 17 at week 12, and 47 vs. 27 at week 25. For seven patients, tumor volume at week 0 could not be determined, due to a scattered response in the area of the original tumor bed. Within clustering analyses, miRNA expression was also compared against pathological complete response (pCR), which is defined according to RECIST criteria as the disappearance all invasive disease in the breast and lymph nodes.

### 2.3 miRNA expression profiling

Total RNA from the tumor samples was extracted using the TRIzol reagent (Invitrogen, Oslo, Norway), according to the manufacturer's protocol. The Agilent Human miRNA Microarray V21.0 (Agilent, Santa Clara, CA, USA; 8 × 60 K) with design ID 070156 was used to profile miRNA expression. For each sample, 150 ng of RNA was amplified and hybridized on the array. Data were required to pass stringent QC parameters established by Agilent that included tests for excessive background fluorescence, excessive variation among probe sequence replicates on the array, and measures of the total gene signal on the array to assess low signal. Samples failing to meet quality standards were relabeled, hybridized to arrays, and rescanned. If a sample failed QC assessment a second time, the sample was excluded from the analysis. Scanning was performed on Agilent Scanner G2565A, and signals were extracted with feature extraction v9.5 (Agilent, Santa Clara, CA, USA). Microarray data are available in the ArrayExpress database (http://www.ebi.ac.uk/arrayexpress) under accession number E‐MTAB‐7634.

### 2.4 Statistical analyses

All statistical analyses were performed in r version 3.1.2 (R Core Team, 2018). For each sample, the miRNA expression values were centered to the median of the observed values. Then, all miRNAs expressed in < 80% of the samples were removed. Subsequent analyses were based on the remaining 627 expressed miRNAs. In order to identify patient‐ and miRNA clusters based on miRNA expression, hierarchical clustering with average linkage and Pearson correlation distance was used. To determine the number of distinct clusters and assign samples to clusters, the Partitioning Algorithm using Recursive Thresholding (PART) (Nilsen *et al.*, 2013) was used, using the ‘clusterGenomics’ package in r. Fisher's exact test was applied to assess associations between categorical clinical variables and cluster assignment. To compare miRNA expression values in two subgroups of tumors, a two‐sample Wilcoxon test was used. All miRNAs with false discovery rate (FDR) < 5% according to the Benjamini–Hochberg (BH) procedure were considered as significant. All, except one sample from week 25, had both mRNA and miRNA expression values and these were used further to calculate miRNA–mRNA Pearson correlations and associated *P*‐values. All miRNA–mRNA pairs with Bonferroni‐adjusted *P*‐value ≤ 0.05 and absolute correlation value above 0.3 were considered as significant. Gene expression data are available in the ArrayExpress database (http://www.ebi.ac.uk/arrayexpress) under accession number E‐MTAB‐4439.

To examine the global pattern of associations between miRNA and protein expression, protein expression was modeled as a function of powers of mRNA in‐*cis* and miRNA expression (Aure *et al.*, 2015). The model allows a range of functional dependencies between miRNAs and proteins and adjusts for the effect of mRNA expression on protein expression.

Venn diagrams for comparison of overlapping miRNAs were generated using the Venny tool (Oliveros, 2007–2015).

### 2.5 Pathway analyses

Functional annotation and pathway analysis were performed using ingenuity pathway analysis (IPA; Ingenuity Systems, Redwood City, CA, USA) on genes significantly correlated to selected miRNAs of interest. Canonical pathways enriched among the genes in each list were identified in IPA using a Fisher's exact test. Pathways with a FDR < 5% according to the Benjamini–Hochberg procedure were further considered.

### 2.6 Predicting target genes for miRNAs

TargetScan Human version 7.0 (Friedman *et al.*, 2009; Grimson *et al.*, 2007; Lewis *et al.*, 2005) was employed to predict target genes for candidate miRNAs. This database uses an algorithm based on conservation criteria and was ranked as the most robust compared to several other databases in a previous study (Baek *et al.*, 2008). Predictions were filtered for transcripts with context score no larger than −0.35, where the targets with lowest context scores represent the most confident targets. To improve reliability of the target genes predicted, a second target prediction database, DIANA‐microT‐CDS (Maragkakis *et al.*, 2009; Paraskevopoulou *et al.*, 2013) was also employed. In this algorithm, conservation is a feature and not a filter, which increases sensitivity to miRNA targets that are lineage specific. Here, low confidence targets were eliminated by filtering out targets with a total context score above or equal to 0.7. Potential targets for significantly up‐ and downregulated miRNAs were retrieved and matched to the actual regulation of the gene.

## 3 Results

### 3.1 Clustering of miRNA expression from pretreatment biopsies identified patient subgroups associated with treatment response

**Figure 1:**
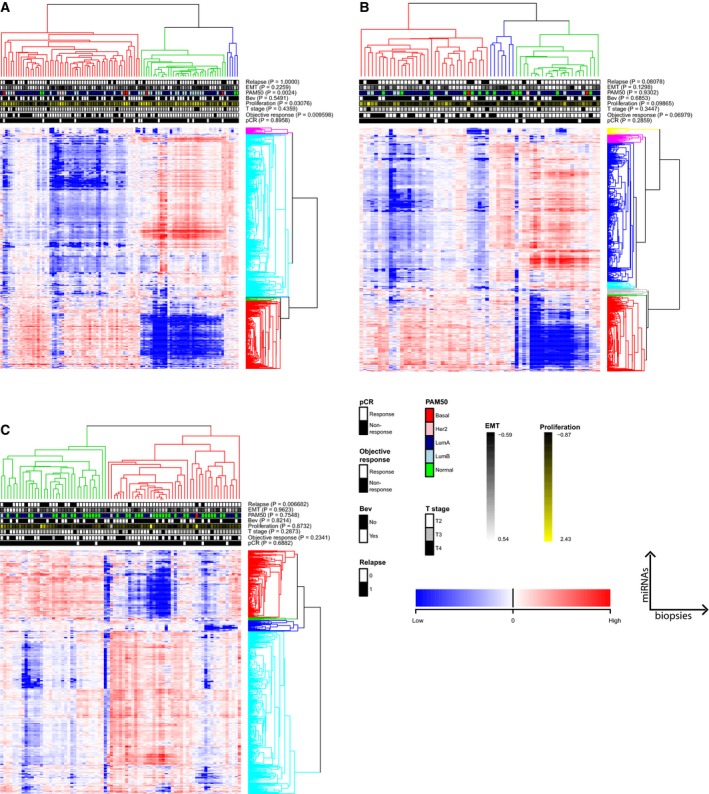
Unsupervised hierarchical clustering of miRNA expression from all patients with biopsies before treatment (week 0; A); after 12 weeks of treatment; (B) and at the end of treatment (week 25); (C). miRNAs are shown in rows and patients in columns. Clusters were identified using the PART method (Nilsen, *et al.*, 2013) with Pearson correlation‐based distance and average linkage. *P*‐values for associations between identified clusters and patient clinical parameters based on Fisher`s exact tests are indicated. Patients clustered significantly according to OR, proliferation, and PAM50 subtypes at week 0, while only relapse was significantly associated with patient clustering at week 25.

Hierarchical clustering of miRNA expression of all biopsies obtained before treatment (week 0) identified three clusters with OR separating significantly from nonresponders (non‐OR; Fisher's exact test, *P* = 0.0096, Fig. 1A). Importantly, patients were not stratified on treatment arm in this analysis. A significant association was seen between the three clusters and PAM50 subtypes as well as proliferation score, with a trend toward association between objective responders (ORs), Luminal B (LumB) subtype, and high proliferation score (Fig. 1A). These associations observed at time point 0 were not seen at week 12 and week 25 (Fig. 1B and C); at week 12 tumors clustered with no significant associations to any of the clinical variables (Fig. 1B). Notably, at operation time at week 25 patients clustered significantly according to relapse (Fig. 1C).

Several of the miRNAs present in the five clusters in Fig. 1A seemed to consistently cluster together (Table S1) throughout the course of treatment (Fig, 1B,C, cluster colors were arbitrarily assigned). This was true for the second largest cluster in Fig. 1A (colored red in Fig. 1A–C), where 73.8% of the miRNAs were present in this cluster at all time points (Table S1). In contrast, for the largest cluster in Fig. 1A (colored turquoise in Fig. 1A), only 17% of the miRNAs were present in this cluster at all time points. Nevertheless, 89% of the miRNAs in the latter cluster clustered together at week 0 and week 25.

We performed a time course study of miRNA expression also for the patients where biopsies were available from all three time points (*n* = 48; Fig. S1). Only two clusters were identified in this analysis, resembling the two main clusters found in Fig. 1A. In addition to the significant association to OR and tumor stage at week 0 in this restricted set, patient clustering showed a borderline significant trend toward association to proliferation (*P* = 0.057) and the clustering pattern changed similarly from week 0 to week 25.

When stratifying according to treatment arm, miRNA expression profiles before treatment initiation could not identify any miRNAs with differential expression between OR and nonresponders (non‐OR; BH‐corrected *P*‐value < 0.05).

### 3.2 Multiple cancer‐associated miRNAs were deregulated in responders before treatment initiation

We then focused on the miRNAs that contributed to the clustering and the association to response in the patients from both treatment arms. Wilcoxon analysis resulted in 217 miRNAs with significant differential expression between OR (*n* = 68) and non‐OR (*n* = 22) before treatment initiation (Table S2). Of these, 71 miRNAs were upregulated and 146 downregulated in OR vs. the non‐OR. Among the downregulated miRNAs were members of the let‐7 family (let‐7f‐5b, 7e‐5p, 7a‐5p, let‐7d, let7g) and the miR‐200 family (miR‐200a‐3p, miR‐200b‐3p, and miR‐429). Both of these families regulate the expression of multiple genes related to EMT and metastasis (Ding, 2014). In addition, miRNAs known as tumor suppressors, like miR‐125a/b (Yan *et al.*, 2018), and onco‐mirs, like miR‐21 (Yan *et al.*, 2011), were also found significantly downregulated in ORs at week 0 compared to non‐ORs.

Interestingly, 55 of the 71 (77.5%) upregulated miRNAs had nomenclature numbers > 3000. Only eight out of the 146 downregulated miRNAs (5.5%) had similar high numbers. According to the miRNA annotation criteria (Ambros *et al.*, 2003), the miRNA identifying numbers are assigned sequentially according to discovery. Thus, the biological functions of miRNAs with high nomenclature are less characterized and studied due to their more recent discovery.

### 3.3 Treatment‐induced changes in miRNA expression over time are linked to decreased proliferation and increased chemotherapy sensitization, with a stronger effect in the bevacizumab arm

**Figure 2:**
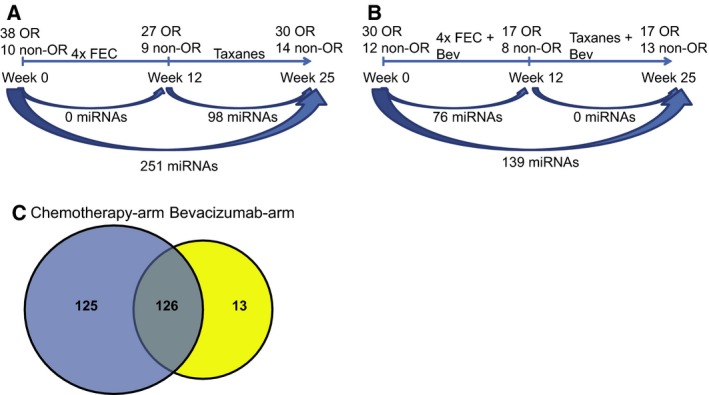
Changes in miRNA expression over time, regardless of treatment response, within (A) the chemotherapy arm and (B) the Bev arm. (C) 126 miRNAs were found differentially expressed in both treatment arms from week 0 to week 25, while 125 miRNAs were exclusively differentially expressed in the chemotherapy arm. Thirteen miRNAs were differentially expressed only in the Bev arm.

We then looked at the numbers of significantly differentially expressed miRNAs (BH‐corrected *P*‐value < 0.05) between time points, that is, from treatment initiation (week 0) until time of operation (week 25) in each treatment arm. A larger number of miRNAs were deregulated in the chemotherapy arm (*n* = 251 miRNAs) compared to the Bev arm (*n* = 139 miRNAs; Fig. 2A,B and Table S3A,B, respectively), regardless of treatment response. However, the two treatment arms differed in the time interval wherein the greater change in expression occurred. While in the Bev arm, a larger number of deregulated miRNAs were observed already at the early interval, a higher number of miRNAs were deregulated at the later interval in the chemotherapy arm. In the biopsies from the patients in the Bev arm, we found 76 miRNAs changing from week 0 to week 12, compared to no miRNAs at the later interval (week 12–week 25), while in the chemotherapy arm there were 98 miRNAs that changed significantly between week 12 and 25, compared to no miRNAs at the early time interval. We were interested in whether the change in miRNA expression in the Bev arm could be explained by Bev having a chemotherapy‐enhancing effect, or whether Bev elicits molecular effects on the miRNA expression that differ from those induced by chemotherapy. Therefore, the 139 differentially expressed miRNAs from week 0 to week 25 in the Bev arm were compared to the 251 differentially expressed miRNAs from week 0 to week 25 in the chemotherapy arm. As illustrated in Fig. 2C and Table S3C, 126 miRNAs were found overlapping between the two treatment arms across the treatment weeks, regardless of response to therapy. All of these were regulated in the same direction in both treatment arms; 120 miRNAs were upregulated and 6 miRNAs downregulated. Interestingly, several of the miRNAs with > 3‐fold upregulation in both treatment arms from week 0 to week 25 (Table 1) such as miR‐100‐5p and miR‐125b are known to inhibit proliferation, migration, and invasion in BC cell lines and tissue (Gong *et al.*, 2015; Li *et al.*, 2018). Furthermore, several of these miRNAs such as miR‐328 and miR‐195 have been reported to sensitize breast tumors to chemotherapy (Pan *et al.*, 2009; Yang *et al.*, 2013).

**Table 1:** Differentially expressed miRNAs from week 0 to week 25 with more than threefold upregulation in both treatment arms and with demonstrated roles in BC tissue and cell lines.

miRNA^a^	Functional role	Identified target gene	Reference
miR‐99a‐5p	Suppress proliferation and migration	IGF‐1R	Xia *et al. *(2016)
miR‐125b‐5p/ miR‐125b‐2‐3p	Inhibits BC proliferation, migration, and invasion/regulates cisplatin resistance in BC.	KIAA1522/HAX1	Hui *et al. *(2018), Li *et al. *(2013)
miR‐187‐5p	Increases aggressiveness and invasion		Mulrane *et al. *(2012), Mandujano‐Tinoco *et al. *(2017)
miR‐100‐5p	Suppress proliferation, migration, and invasion	FZD‐8 and Wnt/β‐catenin pathway	Gong *et al. *(2015), Jiang *et al. *(2016)
miR‐143‐3p/5p	Inhibits invasion and proliferation, induces hypermethylation	DNMT3A	Ng *et al. *(2014), Johannessen *et al. *(2017)
miR‐377‐3p	Lower expression in HER2+ vs. HER2− BC		Leivonen *et al. *(2014)
miR‐195‐3p/5p	Inhibits proliferation, invasion, metastasis, and angiogenesis/ Increases adriamycin sensitivity	FASN, HMGCR, ACACA, CYP27B1, IRS1, Raf‐1	Yang *et al. *(2013), Singh *et al. *(2015), Wang *et al. *(2016)
miR‐328‐5p	Inhibits proliferation and increases drug sensitivity	RAGE, BCRP	Pan *et al. *(2009), Luo *et al. *(2018)
miR‐497‐5p	Inhibits growth, migration, invasion, and EMT	Cyclin E1, Raf‐1, Ccnd1, Slug	Li *et al. *(2011), Luo *et al. *(2013), Wu *et al. *(2016)
miR‐376a/c‐3p	Inhibits growth	NRP‐1	Zhang *et al. *(2018)
miR‐145‐5p	Inhibits proliferation and migration	TGF‐β1, FSCN‐1	Zhao *et al. *(2016), Ding *et al. *(2017)
miR‐381‐3p	Inhibits proliferation, cell cycle progression, and migration/increases doxorubicin sensitivity	SETDB1, FYN	Mi *et al. *(2018), Wu *et al. *(2018)
miR‐4472	Downregulated in chemoresistant BC tissue		Wang *et al. *(2018b)

a BH‐adjusted *P*‐values ≤ 0.05.

In contrast to the most upregulated miRNAs found in patients from both treatment arms, all but one of the 13 miRNAs (miR‐125a‐3p) that changed from week 0 to week 25 exclusively in the Bev arm were downregulated (Table 2). Importantly, the majority of the downregulated miRNAs have been identified as oncogenic miRNAs, while the upregulated miR‐125a‐3p has been identified as a tumor suppressor in several cancer forms, including BC (Alzrigat and Jernberg‐Wiklund, 2017; Xu *et al.*, 2016).

**Table 2:** Differentially expressed miRNAs from week 0 to week 25 found exclusively in the bevacizumab arm.

miRNA^a^	Fold change	Functional role	Identified target gene	Reference
miR‐183‐5p	0.28	Promotes proliferation and migration		Li *et al. *(2014) Macedo *et al. *(2017)
miR‐1307‐5p	0.45	Regulates cisplatin resistance in BC	MDM4	Wang and Zhu (2018)
miR‐1260a	0.51	Unknown in BC		
miR‐200c‐3p	0.53	Suppression of BC migration, invasion, and metastasis	FHOD1, PPM1F, Foxf2	Jurmeister *et al. *(2012), Zhang *et al. *(2017)
miR‐181c‐3p	0.54	Promotes proliferation	PTEN	Zhang and Zhang (2015)
miR‐1181	0.63	Inhibits invasion and proliferation in pancreatic cancer	STAT3	Wang *et al. *(2017)
miR‐7977	0.67	Unknown in cancer		
miR‐181b‐5p	0.67	Promotes EMT and chemoresistance in BC	YWHAG, Bim	Yoo *et al. *(2016) Zheng *et al. *(2016)
miR‐1260b	0.71	Overexpression in prostate cancer		Said *et al. *(2018)
miR‐93‐5p	0.72	Increases chemotherapy resistance	Bcl‐2, P‐gp, PTEN	Chu *et al. *(2017), Li *et al. *(2017), Wang *et al. *(2018a)
miR‐4261	0.74	Increases proliferation and migration of CRC	MCC	Jiao *et al. *(2017)
miR‐106b‐5p	0.79	Increases chemotherapy resistance	PTEN, SMAD7, EP300	Hu *et al. *(2016), Li *et al. *(2017)
miR‐125a‐3p	1.33	Tumor suppressor and potentiates chemotherapy resistance	HuR, BAP1	Guo *et al. *(2009), Xu *et al. *(2016), Yan *et al. *(2018)

a BH‐adjusted *P*‐values ≤ 0.05.

Altogether increased expression of tumor suppressor miRNAs in both treatment arms, while downregulated oncogenic miRNAs in the Bev arm could indicate a general suppression of tumor proliferation and increased sensitivity to chemotherapy over time within the tumors receiving Bev treatment.

We also analyzed the number of significantly differentially expressed miRNAs between time points in each treatment arm only within patients from who we had obtained biopsies from all three time points (regardless of response status). This included 23 ORs and 5 non‐ORs in the chemotherapy arm, and 11 ORs and 7 non‐ORs in the Bev treatment arm. However, none of the miRNAs significantly changed expression over time after BH correction for multiple testing in either of the two treatment arms (data not shown).

### 3.4 miRNAs are highly correlated to histone H3 protein expression throughout treatment

**Figure 3:**
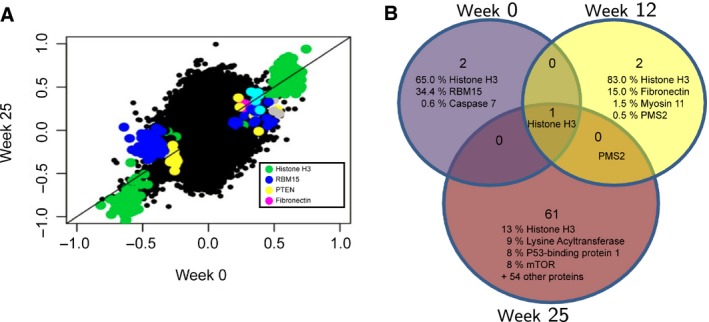
(A) miRNA–mRNA–protein integration model showing the miRNA–protein associations being kept most constant over time. On the *x*‐ and *y*‐axis, the beta value is plotted for a miRNA–protein pair at week 0 (*x*‐axis) and week 25 (*y*‐axis). (B) Venn diagram showing the proteins with most associations to miRNAs at each time point. Percentage indicates the number of associated miRNAs to each protein within the total number of significant miRNA–protein associations. Histone H3 ranked highest with most associations at all three time points.

By combining miRNA expression with mRNA and protein expression, we next attempted to acquire a more integrated and global view of the regulatory circuits that formed in result of the treatment. We therefore combined the expression data from three molecular platforms at three time points in order to gain insight into the combined effect of miRNA and mRNA expression on protein expression over time. To achieve that, protein expression was modeled as a function of mRNA and miRNA expression in a bivariate regression model for each miRNA as described previously (Aure *et al.*, 2015). The model was applied to mRNA and protein expression for 210 selected cancer‐associated genes and the 627 miRNAs studied here. The effect of one miRNA at a time was considered, for all three time points. These analyses were performed for all patients together across treatment arms and response groups. From these analyses, a beta value (indicating the association between a miRNA and a protein) was computed for each time point for all combinations of miRNAs and proteins. From these analyses, significant changes in miRNA expression were seen over time. Interestingly, almost all significantly changing miRNAs were correlated to H3 histone family member 3 (histone H3) across the treatment course (Fig. 3A,B).

Histone H3 was not found differentially expressed in responding patients in either of the two treatment arms from week 0 to week 25, supporting the stable expression of this gene throughout the treatment. Furthermore, as seen in Fig. 3B and Table S4, the number of proteins involved in miRNA–protein association increased from three proteins correlating to 134 miRNAs (histone H3, RBM15, and caspase 6) at week 0 to 63 proteins correlating to 120 miRNAs at week 25. This suggests a broad shift in miRNA–protein correlations throughout the treatment course.

### 3.5 Differentially expressed miRNAs in bevacizumab‐responding patients correlate with proliferation

To see whether the significantly differentially expressed miRNAs in the two treatment arms were linked to treatment response, the patients from each treatment arm were further split into OR vs. nonresponders (non‐OR) and differentially expressed miRNAs were identified between week 0 and 25.

**Figure 4:**
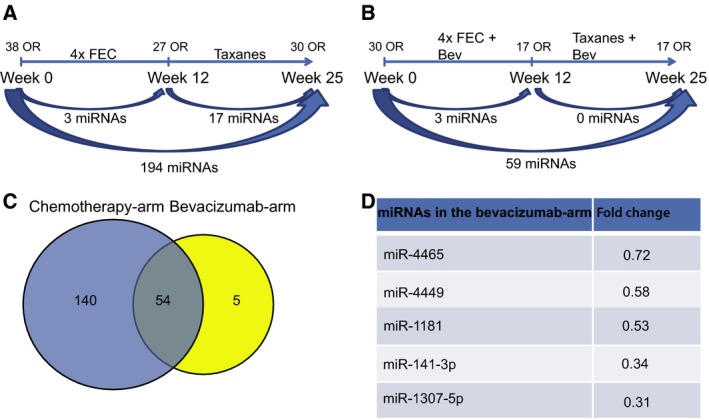
Significant changes in miRNA expression over time in responding patients only within (A) the chemotherapy arm and (B) the Bev arm. (C) 54 miRNAs were found overlapping in the two treatment arms from week 0 to week 25, while 140 miRNAs were found exclusively in the chemotherapy arm. (D) The five miRNAs found exclusively in the Bev arm.

A total of 194 miRNAs were differentially expressed in the responding patients who were given chemotherapy only (Fig. 4A and Table S5A), while only 59 miRNAs were found significantly differentially expressed from week 0 to week 25 in the responders who received Bev in addition (Fig. 4B and Table S5B). As expected, all the 54 overlapping miRNAs found differentially expressed in responders in both treatment arms (Fig. 4C and Table S5C) were also found among the 126 overlapping miRNAs in Fig. 2C, the latter which assessed miRNA expression changes regardless of treatment response.

As previously shown in Silwal‐Pandit *et al. *(2017) and Hoglander *et al. *(2018), Bev treatment was most effective in patients with highly proliferating tumors. Therefore, the differentially expressed miRNAs between OR vs. non‐OR in each treatment arm were correlated to a proliferation score (Parker *et al.*, 2009). Of the 59 differentially regulated miRNAs from week 0 to week 25 in the Bev arm, 44 were significantly correlated to the proliferation score (Bonferroni‐corrected *P* value < 0.05 and absolute Pearson correlation ≥ 0.3) when summarizing all the ER‐positive patients from all time points (Table S6). However, when looking at each time point separately, only eight miRNAs listed in Table 3 were found significantly correlated at week 0. Of these, six miRNAs were significantly negative, while two miRNAs were significantly positive correlated to the proliferation score. Interestingly, the two positive correlating miRNAs were among the five downregulated miRNAs in the Bev arm, suggesting a potential decrease in proliferation in Bev‐responding tumors from week 0 to week 25. Furthermore, miR‐4465, together with miR‐195‐3p, were the only two miRNAs among the 59 Bev‐associated subset that were significantly correlating to proliferation at week 12, while none were kept significant at week 25 (Table S6). In the chemotherapy arm, 26 of the 140 miRNAs correlated significantly (Bonferroni‐corrected *P* value < 0.05 and absolute Pearson correlation ≥ 0.3) to proliferation across all time points, while 28 miRNAs were found significant at week 0 and none at weeks 12 and 25 (data not shown).

**Table 3:** Significantly deregulated miRNAs in bevacizumab‐responding patients and their correlation to proliferation at week 0.

miRNA	NeoAva		Nik‐Zainal *et al*.	
	Correlation^a^	*P*‐value^b^	Correlation^a^	*P*‐value^b^
miR‐145‐5p	−0.53	1.65E−06	−0.21	1.13E‐01
miR‐99a‐5p	−0.48	4.33E‐05	−0.24	2.55E‐02
miR‐4324	−0.47	5.95E‐05	−0.15	1.00E+00
miR‐125b‐2‐3p	−0.47	6.63E‐05	−0.12	1.00E+00
miR‐100‐5p	−0.45	1.72E‐04	−0.23	3.99E‐02
miR‐125b‐5p	−0.45	2.06E‐04	−0.18	4.61E‐01
miR‐1181	0.46	1.32E‐04	0.21	8.74E‐02
miR‐4465	0.46	1.24E‐04	0.33	5.22E‐05

a Pearson correlation.

b BH‐adjusted *P*‐values ≤ 0.05.

To validate the correlation of expressed miRNAs to proliferation, miRNA expression from an independent BC cohort (Nik‐Zainal *et al.*, 2016) was used. In this cohort, proliferation scores were not available; thus, the total number of mitoses in the ER‐positive samples was used as a proxy. Only miRNAs with significant correlation to proliferation in the present dataset were tested. As seen in Table 2, only miR‐4465 showed a significant correlation (Pearson's *r* = 0.3), of the Bev‐associated miRNAs at week 0. Furthermore, as in the NeoAva dataset, this correlation was positive. In the chemotherapy arm, none of the 140 miRNAs had a Pearson correlation value ≥ 0.3 in the validation dataset (data not shown).

### 3.6 Bevacizumab‐specific miRNAs correlate to cell cycle regulation, transcriptional regulation, and leukocyte extravasation

**Figure 5:**
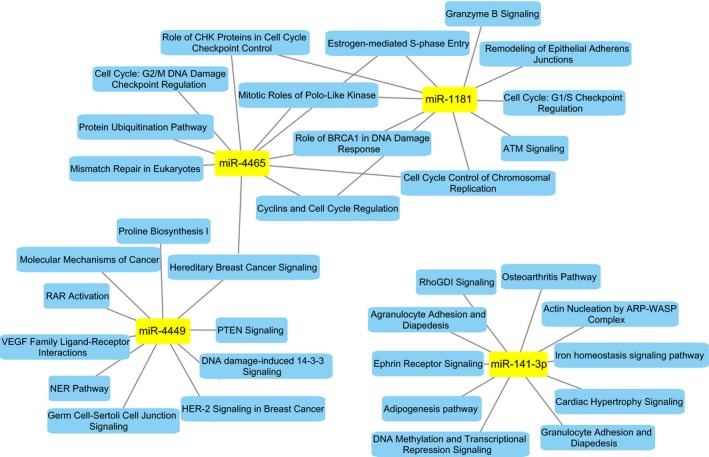
The 10 most significantly associated pathways associated with mRNAs with a significant correlation to four of the five miRNAs found differentially expressed between week 0 and week 25 in Bev‐responding patients.

The five miRNAs found exclusively differentially expressed in responding patients in the Bev arm (Fig. 4D) were correlated to the mRNA expression of all genes in the same tumors. This gave rise to 5409 significant correlations (Bonferroni‐corrected *P*‐value < 0.05) with an absolute correlation value ≥ 0.3 (Table S7). From these, the highest number of correlations was to miR‐4449, miR‐4465, miR‐141‐3p, and miR‐1181 [*n* = 2892 (37.5 %), *n* = 1271 (33 %), *n* = 998 (24 %), and *n* = 246 (5.7 %), respectively], whereas only two transcripts were found significantly correlated to miR‐1307‐5p. The significantly correlating genes to each of the five miRNAs were analyzed for signaling pathway enrichment through IPA. The top 10 most significant pathways linked to these mRNAs were enriched for three main biological functions; mRNAs correlating to miR‐4465 and miR‐1181 were highly associated with cell cycle regulation and control and DNA damage response and repair. miR‐4495 was associated with growth factor receptor signaling, like VEGF and HER2/HER3, in addition to DNA damage responses like the nucleotide excision repair pathway (Fig. 5 and Table S8). To confirm that the correlating mRNAs were in fact also significantly deregulated in the tumors, Wilcoxon tests were performed using mRNA expression from responding tumors at week 0 vs. week 25 in each treatment arm. From these analyses, we found 3980 significantly deregulated genes (BH‐corrected *P* < 0.05) in the Bev arm (Table S9A) and 4469 deregulated genes in the chemotherapy arm (Table S9B). To investigate whether the significantly correlating genes to each of the five Bev‐associated miRNAs were also predicted target genes, TargetScan and microT‐CDS were employed. As indicated in Table S6, miR‐141‐3p, miR‐4465, and miR‐4449 showed the highest number of correlations to genes that were also predicted targets through either one of the two prediction databases.

miR‐141‐3p was associated with leukocyte extravasation (diapedesis) both within granulocytes (neutrophils, basophils, and eosinophils) and agranulocytes (monocytes and lymphocytes), in addition to migration‐ and adhesion‐associated processes like Ephrin receptor signaling and Actin Nucleation By ARP‐WASP Complex. An interesting predicted target with negative correlation to miR‐141‐3p was *CXCL12* (*SDF‐1*), due to its immune‐associated processes. Gene expression analyses confirmed a significant upregulation of *CXCL12* in objective responding tumors from week 0 to week 25, supporting the potential targeting effect of miR‐141‐3p on this gene.

The majority of correlating and predicted target genes for miR‐141‐3p were involved in DNA binding and transcriptional regulation, like ETS transcription factor (*ERG*), H2A histone family member Z (*H2AFZ*), six homeobox 4 (*SIX4*) signal transducer, and activator of transcription 5B (*STAT5B*) and zinc finger protein 281 (*ZNF281*). This was in line with the pathway ‘DNA methylation and transcriptional repression signaling’ as one of the most significant pathways associated with miR‐141‐3p. Importantly, and in contrast to the other four Bev‐specific miRNAs, none of the pathways linked to miR‐141‐3p passed multiple testing correction (Table S7).

As illustrated in Table 4, many of the correlating and predicted target genes of miR‐4465 were also involved in transcriptional regulation and epigenetically modification of chromatin, including members of the Polycomb group (PcG) proteins, like *EPC1* and *EZH2*. Of special interest was the correlation between miR‐4465 and both *EZH2* and *HMGA1*, as both of these genes were among the genes with highest correlation score, in addition to being predicted targets with high target score in both prediction databases employed. Both of these genes, together with *E2F7* and *HOXA5*, play important roles in regulating gene transcription. Importantly, due to their positive correlation, a direct targeting of miR‐4465 on these transcripts cannot be assumed.

**Table 4:** miR‐4465 correlating genes with both predicted target scores and significant regulation in bevacizumab‐responding patients from week 0 to week 25.

mRNA	Gene name	Correlation to miR‐4465^a^	mRNA fold change in bev‐arm W0 to W25^b^
BID	BH3 interacting domain death agonist	0.46	0.73
PNRC1	Proline‐rich nuclear receptor coactivator 1	−0.35	1.65
C20orf24	Chromosome 20 open reading frame 24	0.48	0.75
HMGA1	High‐mobility group AT‐hook 1	0.38	0.49
EZH2	Enhancer of zeste 2 polycomb repressive complex 2 subunit	0.45	0.38
MEMO1	Mediator of cell motility 1	0.44	0.82
TTC13	Tetratricopeptide repeat domain 13	0.34	0.76
ARPC3	Actin‐related protein 2/3 complex, subunit 3, 21kDa	0.38	0.75
KPNA2	Karyopherin alpha 2 (RAG cohort 1, importin alpha 1)	0.47	0.53
EPC1	Enhancer of polycomb homolog 1 (Drosophila)	−0.34	1.35
HOXA5	Homeobox A5	−0.37	2.13
FLVCR1	Feline leukemia virus subgroup C cellular receptor 1	0.38	0.59
FAM172A	Family with sequence similarity 172, member A	−0.47	1.45
CCDC28A	Coiled‐coil domain containing 28A	−0.35	1.26
DEPDC1B	DEP domain containing 1B	0.49	0.68
E2F7	E2F transcription factor 7	0.46	0.56
CKS2	CDC28 protein kinase regulatory subunit 2	0.45	0.33
UCK2	Uridine–cytidine kinase 2	0.39	0.72
ZNF598	Zinc finger protein 598	0.37	0.80
INTU	Inturned planar cell polarity protein	−0.40	1.45
ARMCX4	Armadillo repeat containing, X‐linked 4	−0.36	1.81
PHF21A	PHD finger protein 21A	−0.44	1.35
PELI2	Pellino E3 ubiquitin protein ligase family member 2	−0.39	2.05
FAM49B	Family with sequence similarity 49, member B	0.44	0.61
IL2RA	Interleukin 2 receptor, alpha	0.32	0.80
DEPDC1	DEP domain containing 1	0.47	0.63

a Pearson correlation and Bonferroni‐adjusted *P*‐value ≤ 0.05

b BH‐adjusted *P*‐values ≤ 0.05.

Interestingly, correlation was found between miR‐4449 and the predicted target gene *ERG*, which besides regulating cell proliferation and differentiation, also plays a crucial role in promoting angiogenesis and vascular stability (Birdsey *et al.*, 2015; Shah *et al.*, 2017). This was in line with the association between this miRNA and the pathway ‘VEGF family ligand‐receptor interactions’ when assessing pathway enrichment of correlated genes.

Altogether, among the correlating and predicted targets of the five Bev‐associated genes, many seem to be linked to DNA binding and transcriptional regulation.

## 4 Discussion

MicroRNAs have emerged as regulators for a range of cellular events like proliferation, differentiation, apoptosis, and metabolism. Studies have demonstrated a role for miRNAs in resistance or sensitization of tumors to chemotherapy and radiotherapy. Previous studies have looked at miRNA´s role as predictors for antiangiogenic therapy by comparing their expression in responders vs. nonresponders either before or after treatment (Kiss *et al.*, 2017), or changes in the level of circulating miRNAs (Hansen *et al.*, 2015). The present study is, to our knowledge, the first study to address the changes in miRNA expression within the tumor over the course of treatment, and their correlation to response.

Here, we show that neoadjuvant addition of Bev to chemotherapy in HER2‐negative, ER‐positive BC tumors elicits many of the same molecular changes as chemotherapy alone. However, the changes occur earlier in the treatment course, compared to the chemotherapy arm. Previous studies have shown that Bev promotes a more homogenous intratumor distribution of paclitaxel, thereby improving the antitumor response (Cesca *et al.*, 2016). Based on these reports, we hypothesize that the addition of Bev to chemotherapy elicits a faster chemotherapeutic tumor response at the molecular level, but that the final net change within treatment arms would perhaps be the same. This could potentially explain the documented increase in PFS when combining Bev with standard chemotherapy in many clinical trials, but a lack of OS benefit (Montero *et al.*, 2012). Importantly, for both treatment arms chemotherapy was switched from FEC100 to taxanes from week 12 to week 25, making it harder to directly compare the two different time periods. We therefore chose to compare biopsies from before treatment initiation with samples taken at time of surgery within the two treatment arms. Regardless of response, we found that a large proportion of the differentially expressed miRNAs from week 0 to week 25 within the two treatment arms were overlapping; a finding which supports our hypothesis of Bev potentiating the chemotherapeutic effect. Furthermore, both the 126 overlapping miRNAs found in both treatment arms and the 13 deregulated Bev‐specific miRNAs from week 0 to week 25, were associated with general suppression of tumor proliferation, and increased sensitivity to chemotherapy. The association toward sensitizing the cells to chemotherapy also supports the hypothesis of Bev exerting its effect through potentiating the effect of the chemotherapeutic drug.

Clustering of miRNA expression before treatment initiation separated the patients according to objective tumor response, proliferation, and PAM50 subtypes. The change in patient clustering and loss of association to clinical parameters from week 0 to week 25 indicates a change in miRNA expression as the tumor evolves or responds to therapy. The loss of association to response and proliferation could be explained by the gradual shift of tumors becoming more normal‐like, as shown in the heatmaps of week 25 and in the previous gene expression analyses (Silwal‐Pandit *et al.*, 2017).

The latter study also showed that patients with highly proliferating tumors and basal‐like subtype responded better to therapy, and that proliferation score was more strongly suppressed in the combination therapy arm. In line with this, we found a correlation between the two Bev‐specific miRNAs miR‐4465 and miR‐1181 and proliferation scores, wherein the former was verified in another clinical BC dataset. Whether the downregulation of miR‐4465 over time merely reflects a loss of the most proliferating tumor cells during the course of treatment, or whether a direct or indirect mechanism between this miRNA and proliferation exists needs further studies. As miR‐4465 is newly introduced into the Agilent microarray platform, we did not find additional clinical cohorts with matching conditions to verify the findings. However, few patients in the The Cancer Genome Atlas cohort expressed this miRNA (data not shown), which could suggest that miR‐4465 reflects a cell type‐specific miRNA.

To gain insight into the combined effect of miRNA and mRNA expression on protein expression over time, protein expression was modeled as a function of miRNA and mRNA expression. Although these analyses are limited to only 210 proteins, the model could highlight some important associations between miRNA expression and gene/protein expression. In these analyses, we found that most miRNAs were associated with histone H3 at all three time points. However, in contrast to week 0 and week 12 wherein almost all expressed miRNAs were linked to this protein, tumors from week 25 showed a greater diversity between miRNAs correlating to a larger set of proteins. Histone H3 represents a replication‐independent histone gene and has been implicated in the epigenetic regulation of diverse biological processes. Incorporation of histone H3 into a nucleosome has shown to accumulate modifications associated with gene activation or open chromatin (Loyola and Almouzni, 2007; McKittrick *et al.*, 2004). Furthermore, histone H3‐null mice embryos show reduced cell proliferation and increased cell death (Jang *et al.*, 2015), and its phosphorylated form (phospho‐histone H3) has been suggested to better represent proliferation than Ki67 (Kim *et al.*, 2017). The gradual loss of miRNA correlations to this protein from week 0 to week 25 supports the observed decrease in proliferating tumor cells in the gene expression analyses from the same patients. The concordance between the gene expression analyses and the miRNA results indicates that miRNAs could have the potential to function as predictors of general treatment response.

We found five deregulated Bev‐specific miRNAs that showed a general high correlation to cell cycle and transcriptional regulation. Interestingly, there was a significant enrichment among positively correlating genes for miR‐4465 involved in chromatin‐ and transcriptional regulation, like histone cluster 1 and 2 family members and *E2F* genes, as well as cell cycle‐related pathways, like cell division cycle‐ and cyclin‐dependent genes. Many of the histone family members associated with miR‐4465 are replication‐dependent histones, which are expressed during S‐phase end in a conserved stem‐loop rather than a polyA tail. Downregulation of these genes indicates a suppression of cell cycle progression. miR‐4465 is not confidently annotated but shares seed with the confidently annotated miR‐26‐5p. High‐mobility group AT‐hook 1 (*HMGA1*) and enhancer of zeste 2 (*EZH2*) polycomb repressive complex 2 (*PRC2*) were the only genes predicted as miR‐4465 targets in both target prediction databases employed, with high prediction scores and which showed significant changes in gene expression from week 0 to week 25. *EZH2* is a histone methyltransferase subunit in *PRC2*, which mediates transcriptional repression through histone methylation (Yoo and Hennighausen, 2012). *HMGA1* functions as a regulator of chromatin structure and gene transcription that can transactivate promoters and thereby drive gene expression in response to extracellular and intracellular signals (Reeves, 2001; Thanos and Maniatis, 1995). Both genes have been found upregulated in several tumors and have been associated with a myriad of fundamental cellular processes, like cell cycle progression, differentiation, apoptosis, and DNA repair (Chen *et al.*, 2012; Esposito *et al.*, 2010; Giannini *et al.*, 2000; Mao *et al.*, 2009; Pawlyn *et al.*, 2016; Schuldenfrei *et al.*, 2011; Ueda *et al.*, 2007; Wu *et al.*, 2011; Zhang *et al.*, 2014). Both miR‐4465 and miR‐26‐5p have been shown to suppress tumor proliferation and metastasis in several cancers by directly targeting *EZH2* and *HMGA1* (Lin *et al.*, 2013; Sun *et al.*, 2017; Zhou *et al.*, 2017). However, the positive correlation between miR‐4465 and both EZH2 and *HMGA1* in this study, also reflected through the downregulation of both the miRNA and genes, contradicts the general belief of miRNAs as inhibitors of gene expression. The positive correlation found here is potentially the result of other regulatory mechanisms, as *EZH2* and *HMGA1* are regulated through several other mechanisms.

The additional finding of miR‐141‐3p correlating to lymphocyte extravasation is in line with previous findings on gene expression (Silwal‐Pandit *et al.*, 2017) and serum cytokines (Jabeen *et al*., 2018), demonstrating high expression of immune‐related genes in patients with pCR to Bev. Importantly, in the latter study ER‐positive and ER‐negative tumors were combined when analyzing patients with pCR vs. non‐pCR. When we performed miR‐141‐3p correlation to all mRNAs within both ER‐positive and ER‐negative patients, we also found a strong and significant link to several immune‐related processes (data not shown). These results may indicate that miR‐141‐3p may play a larger role in the ER‐negative subset of patients, in line with a higher frequency of tumor infiltrating lymphocytes in ER‐negative vs. ER‐positive tumors (Hammerl *et al.*, 2018). Nevertheless, the significant anticorrelation between miR‐141‐3p and *CXCL12* (*SDF‐1*), as well as the latter being a predicted target for this miRNA, does suggest immunological effects from this miRNA in the ER‐positive subset of patients responding to Bev as well. The significant anticorrelation found here has also been validated in other studies (Periyasamy‐Thandavan *et al.*, 2018). The association between the antiangiogenic drug Bev and the downregulation of miR‐141‐3p is also interesting, as *CXCL12* and its receptor *CXCR4* has been identified as critical mediators for ischemia‐specific recruitment of circulating progenitor cells for neovascularization (Ho *et al.*, 2010). In addition, *CXCL12* has been shown to be angiogenic in various tumors (Koshiba *et al.*, 2000; Kryczek *et al.*, 2005). Of note, miR‐141‐3p has been found to be expressed in mammary epithelial cells themselves (McCall *et al.*, 2017).

## 5 Conclusion

In this study, we have demonstrated a difference in miRNA expression between OR and nonresponders at diagnosis (Fisher's exact test, *P* = 0.0096). Notably, after the neoadjuvant treatment (at week 25), patients clustered significantly according to relapse, revealing a set of miRNAs in a tumor sample at operation after treatment that may indicate relapse. In this longitudinal study, the results based on repeated tumor sampling indicate a treatment‐induced deregulation of several miRNAs associated with tumor suppression in responding patients. This was found in both treatment arms over time; however, the earlier change in the combination arm indicates that Bev adds to the effect of the chemotherapeutic drug. Furthermore, the association between Bev‐specific miRNAs and genes involved in proliferation, cell cycle regulation transcriptional control supports an additive effect of antiangiogenic treatment to the more aggressive tumors. The enrichment of immune‐related processes associated with miR‐141‐3p supports our gene expression data, and the potential role of Bev in promoting response in immune activated tumors. Finally, the results demonstrate that miRNA expression analyses add an important layer of information to the understanding of molecular functions in response to antiangiogenic therapy.

## Conflict of interest

The study was cosponsored by Roche Norway and Sanofi‐Aventis, contributing to the funding of the clinical study. No other potential conflicts of interest were disclosed among the authors.

## Author contributions

OE and ALBD designed the study. EML, MRA, OCL, DN, and MHH performed the computations and statistical analyses. EML ran the Agilent miRNA arrays and wrote the paper with input from all authors.

## Supporting information


**Fig. S1.** Unsupervised hierarchical clustering of miRNA expression from the 48 patients with biopsies from all three time points. Clusters and statistics are based on miRNA expression on week 0, and clusters are kept for week 12 and week 25. A significant association was identified between the two clusters, OR and tumor stage (T stage).

**Table S1.** miRNAs present within each of the clusters illustrated in Fig. 1.
**Table S2.** Significantly differentially expressed miRNAs between OR and non‐responders at week 0, regardless of treatment arm.
**Table S3.** (A) Significantly differentially expressed miRNAs from week 0 to week 25 in the chemotherapy arm, regardless of treatment response. (B) Significantly differentially expressed miRNAs from week 0 to week 25 in the Bev arm, regardless of treatment response. (C) Significantly differentially expressed miRNAs from week 0 to week 25 in both treatment arms, regardless of treatment response.
**Table S4.** Bivariate regression model wherein protein expression was modeled as a function of powers of mRNA in‐*cis* and miRNA expression. The model was applied to mRNA and protein expression for 210 selected cancer‐associated genes and the 627 miRNAs studied here. The effect of one miRNA at a time was considered, for all three time points. The beta value indicates the association between a miRNA and a protein.
**Table S5.** (A) Significantly differentially expressed miRNAs between week 0 and week 25 in responding patients in the chemotherapy arm. (B) Significantly differentially expressed miRNAs between week 0 and week 25 in responding patients in the Bev arm. (C) Significantly differentially expressed miRNAs from week 0 to week 25 in both treatment arms, regardless of treatment response.
**Table S6.** Correlations between proliferation score and significantly differentially expressed miRNAs between week 0 and week 25 in Bev‐responding patients.
**Table S7.** Correlations between mRNAs and significantly differentially expressed miRNAs between week 0 and week 25 in Bev treated responders.
**Table S8.** Ingenuity pathways derived from genes with significant correlation to the five Bev‐associated miRNAs.
**Table S9.** (A) Significantly differentially expressed mRNAs between week 0 and week 25 within responding patients in the Bev arm. (B) Significantly differentially expressed mRNAs between week 0 and week 25 within responding patients in the chemotherapy arm.

## Data Availability

Data from the miRNA microarray analyses performed in this study are available in the ArrayExpress database (http://www.ebi.ac.uk/arrayexpress) under accession number E‐MTAB‐7634. Additional gene expression data from the same clinical study are also available in the ArrayExpress database under accession number E‐MTAB‐4439.
