# Supplementary figures and images for: miRNA expression changes during the course of neoadjuvant bevacizumab and chemotherapy treatment in breast cancer
Source: Mol Oncol. 2019 Aug 28;13(10):2278–96. doi: 10.1002/1878-0261.12561 (PMC6763780; doi:10.1002/1878-0261.12561)

Supplementary figure 1:

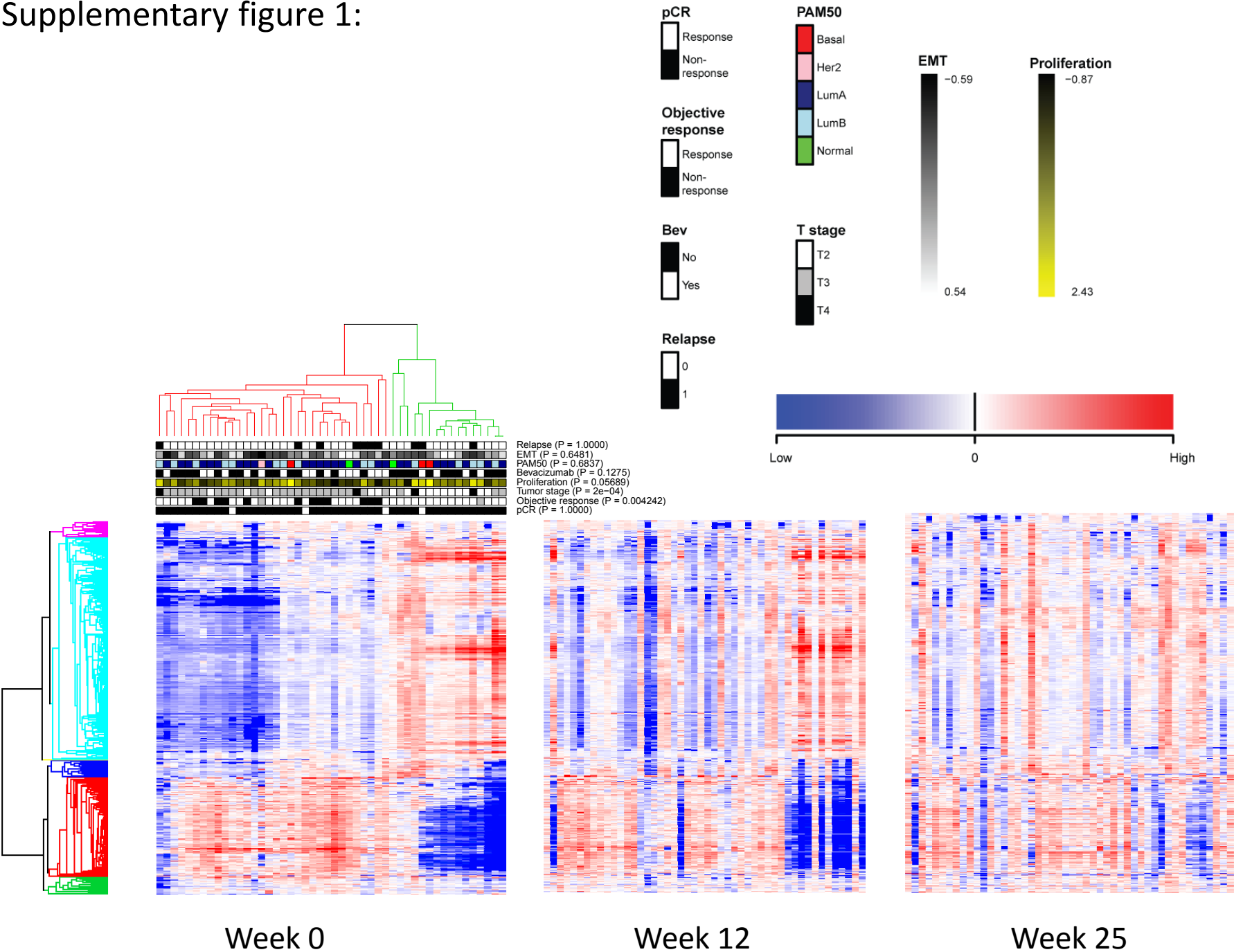

Supplement: Supplementary file 1 — Fig. S1. Unsupervised hierarchical clustering of miRNA expression from the 48 patients with biopsies from all three time points. Clusters and statistics are based on miRNA expression on week 0, and clusters are kept for week 12 and week 25. A significant association was identified between the two clusters, OR and tumor stage (T stage). [file MOL2-13-2278-s001.pdf]
